# MicroRNA based Prediction of Posthepatectomy Liver Failure and Mortality Outperforms Established Markers of Preoperative Risk Assessment

**DOI:** 10.1245/s10434-025-17528-x

**Published:** 2025-06-05

**Authors:** Anna Emilia Kern, David Pereyra, Jonas Santol, Markus Ammann, Sarang Kim, Felix Xaver Huber, Jeremias Weninger, Sarah Brunner, Valerie Laferl, Yannic Herrmann, Anna Jankoschek, Gregor Ortmayr, Benedikt Rumpf, Marcel Schuetze, Rosmarie Valenta, Christian Krestan, Guenther Zauner, Melanie Zechmeister, Susanna Skalicky, Matthias Hackl, Alice Assinger, Thomas Gruenberger, Patrick Starlinger

**Affiliations:** 1 Medical University of Vienna, Vienna, Austria; 2 Department of General Surgery, Division of Visceral Surgery, Medical University of Vienna, Vienna, Austria; 3 Department of Surgery, HPB Center, Vienna Health Network, Clinic Favoriten and Sigmund Freud Private University, Vienna, Austria; 4 Department of Surgery, Division of Hepatobiliary and Pancreas Surgery, Mayo Clinic, Rochester, MN USA; 5 Institute of Vascular Biology and Thrombosis Research, Center for Physiology and Pharmacology, Medical University of Vienna, Vienna, Austria; 6 Department of Surgery, State Hospital Wiener Neustadt, Wiener Neustadt, Austria; 7 Center for Cancer Research, Medical University of Vienna, Vienna, Austria; 8 Department of Surgery, Hospital Barmherzige Schwestern, Vienna, Austria; 9 Department of Diagnostic and Interventional Radiology, Vienna Health Network, Clinic Favoriten, Vienna, Austria; 10 DWH GmbH, Vienna, Austria; 11 Institute of Information Systems Engineering, TU Wien, Vienna, Austria; 12 Institute of Statistics and Mathematical Methods in Economics, TU Wien, Vienna, Austria; 13 TAmiRNA GmbH, Vienna, Austria; 14 Center for Physiology and Pharmacology, Medical University of Vienna, Vienna, Austria

## Abstract

**Background:**

Posthepatectomy liver failure (PHLF) continues to be the most significant factor-determining outcome after hepatic resection, accounting for nearly half of postoperative mortality. In this study, we evaluated whether a newly developed commercially available test measuring circulating microRNAs (miRs) could predict PHLF and compared it with other established liver function tests.

**Patients and Methods:**

A total of 329 patients undergoing liver resection were included and postoperative outcome was assessed. Our previously described P-score, calculated on the basis of three circulating microRNAs (miR-122-5p, miR-192-5p, miR-151a-5p) using the hepatomiR^®^ CE-IVD test, was evaluated and compared with other predictors of PHLF, namely indocyanine green (ICG)-clearance as well as the combined aspartate aminotransferase (AST)-to-platelet ratio index (APRI) and albumin–bilirubin grade (ALBI) score.

**Results:**

Compared with both other liver function tests, P-scores were superior in predicting PHLF and PHLF grades B and C (PHLF B + C) (PHLF B + C: hepatomiR^®^ AUC = 0.835, APRI + ALBI AUC = 0.807; retention rate at 15 min (R15) AUC = 0.690; plasma disappearance rate (PDR) AUC = 0.691). We also documented a superior positive (77%) and negative predictive value (> 90%) for PHLF, along with a close association with postoperative overall survival. A health-economic analysis demonstrated the cost-effectiveness of hepatomiR^®^ in terms of life-years gained due to improved patient risk stratification.

**Conclusions:**

The hepatomiR^®^ P-score outperforms established liver function tests utilized in daily clinical practice for predicting PHLF and identifies patients possibly better served with alternative treatments. A health-economic assessment allowed us to demonstrate that optimized preoperative risk-assessment leads to a cost-effective improvement in patient outcomes.

**Supplementary Information:**

The online version contains supplementary material available at 10.1245/s10434-025-17528-x.

Despite the improvement of surgical techniques and perioperative management of patients undergoing hepatic resection,^1^ posthepatectomy liver failure (PHLF) remains a clinical challenge in oncologic surgery. It occurs in 5–20% of patients, with varying incidence depending on tumor type, extent of resection, and underlying chronic liver disease. Correspondingly, the main cause of short-term postoperative mortality is directly linked to PHLF.^2–5^ This form of hepatic decompensation poses a particular challenge as no causal therapy exists to treat or prevent it.^6,7^ Subsequently, the highest priority remains to reliably assess preoperative risk of PHLF in patients considered for hepatic resection and possibly optimizing individual circumstances.

Here, volumetry of the future liver remnant (FLR) is one of the pillars for adequate preoperative assessment.^8^ Together with dynamic liver function tests such as indocyanine green (ICG)-clearance,^9,10^ individuals undergoing hepatic resections are evaluated for the risk of PHLF. Moreover, the combined aspartate aminotransferase (AST)-to-platelet ratio index (APRI) and albumin–bilirubin grade (ALBI) (APRI + ALBI)-score has been documented to be equally predictive of clinically significant PHLF as ICG-clearance in a multivariable model.^11^ However, even with the development of portal vein embolization (PVE) alone or in combination with ipsilateral liver vein occlusion (PVE/LVD) to enhance the FLR,^12–14^ patients with normal liver function and sufficient liver volume still develop PHLF.

This problem signifies the need for new, innovative biomarkers for preoperative risk assessment. Accordingly, these markers should be noninvasive, highly standardized, and easy to integrate into clinical routine. New testing systems need to be able to identify patients at high risk for PHLF rather than just detecting patients at low risk. Previously, we identified three circulating microRNAs (miRs), namely miR-122-5p, miR-192-5p, and miR-151a-5p, which, when combined, formed the hepatomiR^®^ P-score (P-score) and subsequently predicted PHLF prior to surgery.^15^

The aim of this study was to validate our previous findings regarding the predictive potential of the P-score for PHLF in a large cohort of patients undergoing major and minor liver resection. In addition, their association with other well-established preoperative liver function tests was explored.

## Materials and Methods

The present study was approved by the Institutional Ethics Committee of the Medical University of Vienna, Vienna, Austria (EK#2032/2013), and written informed consent was obtained from all participants.

Further details on methodology, definitions of outcome parameters, and statistical analyses can be found in the supplementary material accompanying this manuscript.

### Study Population

For this study, 329 patients who underwent hepatic resection between February 2011 and December 2021 at Clinic Favoriten (Vienna, Austria), General Hospital Vienna (Vienna, Austria), and Clinic Landstraße (Vienna, Austria) were retrospectively included from a prospectively maintained biobank. All patients were diagnosed with either metastasized colorectal carcinoma (mCRC), hepatocellular carcinoma (HCC), cholangiocellular carcinoma (CCa), benign liver tumors, or other malignancies. Plasma samples were collected 1 day prior to hepatic resection for each participant. Data on the patients’ ethnicity were not recorded, and as a result, ethnic demographics are not reported in this analysis.

### qPCR Analysis of Circulating miRs

Analysis of circulating miRs and the calculation of the hepatomiR^®^ (TAmiRNA GmbH, Vienna, Austria) P-scores were performed according to the manufacturer’s instructions utilizing real-time quantitative polymerase chain reaction (RT-qPCR).

### Health-Economic Modeling

A cost–utility model was developed to simulate the health and budget impact of determining the P-score prior to performing hepatic resections in patients with HCC, CCa, and mCRC compared with the standard of care. The incidence of HCC, CCa, and mCRC, as well as PHLF, was derived from the literature and Austrian registries. The hepatomiR^®^ P-score was assumed to have a positive predictive value (PPV) of 83% and a negative predictive value (NPV) of 85% based on previously published results.^15^

### Cut-Offs

Patients were stratified by risk cut-offs defined in the previously published study.^15^ There, we identified a low-risk cut-off of P > 0.59, which classified patients at lower risk to develop PHLF, as well as a high-risk cut-off of P > 0.68, which identified patients at the highest risk of developing PHLF.

## Results

### Patient Cohort

A total of 329 patients with mCRC, HCC, CCa, benign liver tumors, and other malignancies underwent major and minor hepatic resection. Data on preoperative liver volumetry were available in 60 patients, of whom 3 had a FLR below 30% and 9 below 40% (Table 1). Among these patients, only one with a FLR below 30% developed PHLF. In total, 17 patients had PVE before undergoing resection. Interestingly, although no significant differences in comorbidities could be documented, none of the patients with renal insufficiency or evidence of pulmonary disease were included in the high-risk groups. In addition, data on the type of metastases and neoadjuvant chemotherapy (nCTx) revealed no significant differences except for treatments with FOLFOXIRI + cetuximab and XELIRI + cetuximab. However, it is worth noting that only two patients were present in each of these groups. Patient demographics stratified by risk cut-offs can be found in Supplementary Table S1 and Table S2.

**Table 1:** Patient demographics of the whole cohort (N = 329)

**Parameter**	Median (range)/ *N* (%)	Parameter	Median (range)/ *N* (%)
**Gender**		**Co-morbidities**	
Male	215 (65.3)	Hypertension	122 (53.7)
Female	114 (34.7)	Type 2 diabetes	40 (17.6)
**Age (years)**	65 (22.9)	Renal insufficiency	15 (6.6)
**Hepatic resection**		Pulmonary disease	18 (7.9)
Minor (< 3 segments)	143 (43.5)	**Morbidity**	
Major (≥ segments)	186 (56.5)	No morbidity	159 (48.3)
Open surgery	240 (92.7)	Grade I	25 (7.6)
MIS	19 (7.3)	Grade II	73 (22.2)
FLR < 30%*	3 (5.0)	Grade III	43 (13.1)
FLR < 40%*	9 (15.0)	Grade IV	10 (3.0)
**Tumor type**		Grade V	19 (5.8)
mCRC	151 (45.9)	**PHLF ISGLS**	
HCC	72 (21.9)	No PHLF	288 (87.5)
CCa	70 (21.3)	ISGLS A	11 (3.3)
Other	18 (5.5)	ISGLS B	9 (2.7)
Benign	18 (5.5)	ISGLS C	21 (6.4)
**Co-factors**		**Postop. stay**	
nCTx	109 (33.1)	ICU (days)	1 (0–56)
PVE	17 (14.2)	Total hosp. (days)	8 (1–117)
Steatosis (%)	5 (0-100)	**mCRC**	
Steatohepatitis	67 (20.4)	Synchrone mets.	76 (67.3)
Intraoperative RBCs	28 (8.5)	Metachrone mets.	37 (32.7)
**Fibrosis**		**nCTx regimen**	
Grade I	110 (33.4)	FOLFOX	5 (4.8)
Grade II	38 (11.6)	FOLFOX + Avastin	15 (14.3)
Grade III	23 (7.0)	FOLFOX + cetuximab	2 (1.9)
Grade IV	27 (8.2)	FOLFOX + panitumumab	4 (3.8)
**Preop. parameters**		FOLFIRI + Avastin	4 (3.8)
hepatomiR^®^ P-scores	0.150 (0.002–0.980)	FOLFIRI + cetuximab	6 (5.7)
PDR (%/mL)	20.0 (7.6–40.0)	FOLFIRI + panitumumab	5 (4.8)
R15 (%)	5.0 (0.3–32.0)	FOLFOXIRI	4 (3.8)
APRI + ALBI	−2.59 (−5.6 to 0.38)	FOLFOXIRI + Avastin	13 (12.4)
Platelets (10^3^/µL)	232 (71–1082)	FOLFOXIRI + cetuximab	2 (1.9)
SB (mg/dL)	0.54 (0.2–20.37)	FOLFOXIRI + panitumumab	1 (0.9)
PT (%)	104 (40–150)	FOLFIRINOX + Avastin	2 (1.9)
AP (U/L)	91 (32–1274)	XELOX	3 (2.9)
GGT (U/L)	61 (9–2457)	XELOX + Avastin	26 (24.7)
AST (U/L)	31 (13–418)	XELOX + cetuximab	4 (3.8)
ALT (U/L)	30 (7–806)	XELOX + panitumumab	3 (2.9)
Albumin (g/L)	41.9 (28.1–74.2)	XELIRI + Avastin	3 (2.9)
		XELIRI + cetuximab	2 (1.9)
		XELIRI + panitumumab	1 (0.9)
		XELIRI + panitumumab	1 (0.9)

*ALT* alanine aminotransferase, *AP* alkaline phosphatase, *APRI + ALBI* aspartate aminotransferase-to-platelet ratio index and albumin**–**bilirubin grade, *AST* aspartate aminotransferase, *CCa* cholangiocellular carcinoma, *FLR* future liver remnant, *FOLFOX* folinic acid/5-fluoruracil/oxaliplatin, *FOLFIRI* folinic acid/5-fluoruracil/irinotecan, *FOLFOXIRI* folinic acid/5-fluoruracil/oxaliplatin/irinotecan, *FOLFIRINOX* folinic acid/5-fluoruracil/irinotecan/oxaliplatin, *GGT* gamma-glutamyl transpeptidase, *HCC* hepatocellular carcinoma, *ICU* intensive care unit**,** *ISGLS* International Study Group of Liver Surgery, *mCRC* metastasized colorectal cancer, *mets.* metastases, *N* number, *nCTx* neoadjuvant chemotherapy, *PDR* plasma disappearance rate, *PHLF* posthepatectomy liver failure, *preop.* preoperative, *postop.* postoperative, *PT* prothrombin time, *PVE* portal vein embolization, *R15* retention rate at 15 minutes, *RBCs* red blood cells, *SB* serum bilirubin, *XELOX* capecitabine/oxaliplatin, *XELIRI* capecitabine/irinotecan**;** * preoperative volumetry was available in 60 patients

### HepatomiR^®^ Shows High Predictive Potential for Clinically Relevant PHLF

**Fig. 1:**
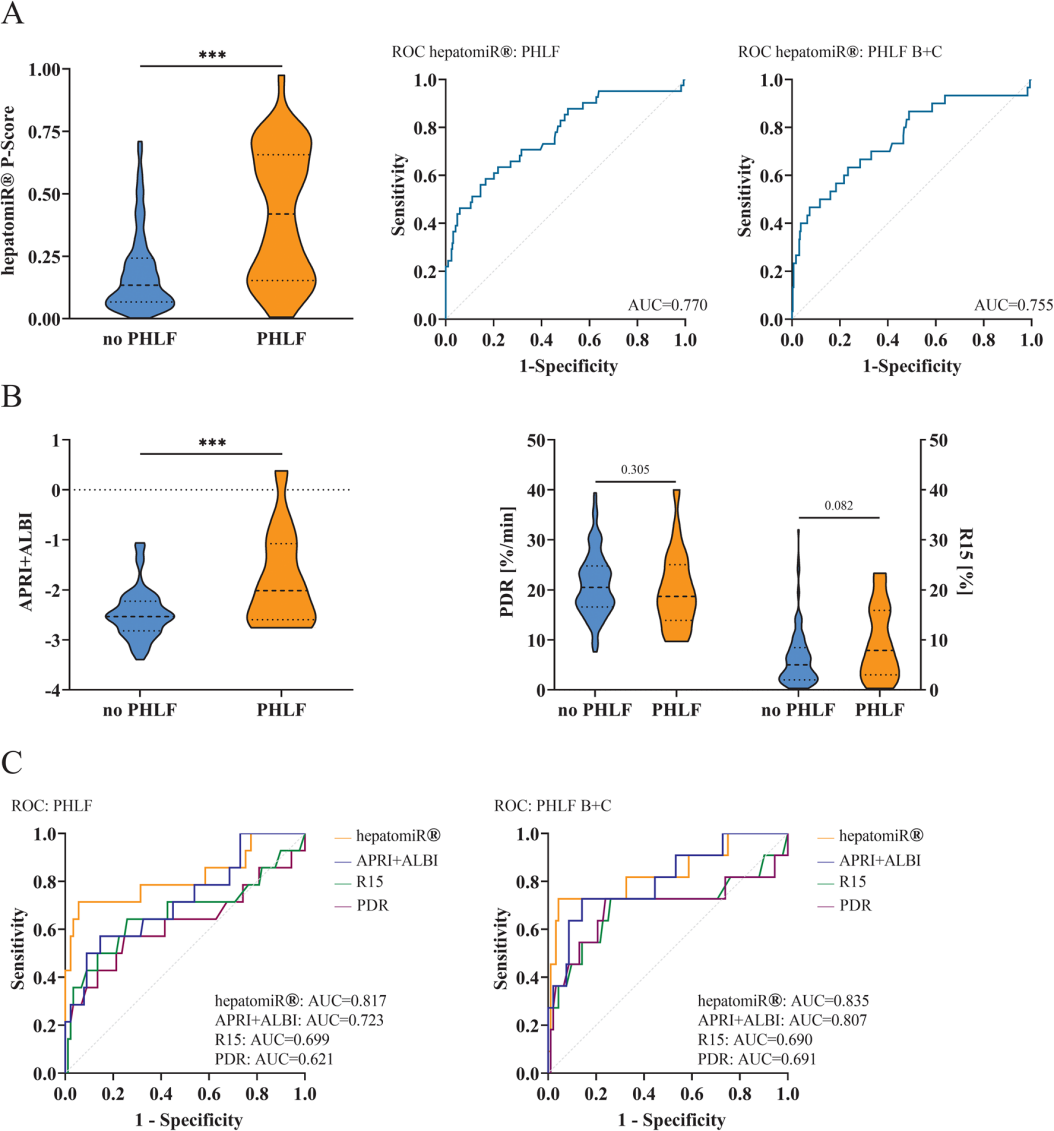
HepatomiR^®^ in the whole cohort and in comparison with other liver function tests: differences of P-scores among patients without and with PHLF, ROC curves comparing the predictive potential of hepatomiR^®^ for PHLF grades A–C and PHLF grade B + C (**A**); differences of APRI + ALBI and ICG-clearance (PDR and R15) in patients without and with PHLF (**B**); ROC curves comparing the predictive potential of hepatomiR^®^, APRI + ALBI, and ICG-clearance (**C**); *APRI+ALBI* aspartate aminotransferase-to-platelet ratio index and albumin-bilirubin grade, *AUC* area under the curve, *ICG* indocyanine green, *PHLF* posthepatectomy liver failure, *PHLF B+C* PHLF grades B and C, *ROC* receiver operating characteristics; * *p* < 0.05, ** *p* < 0.01, *** *p* < 0.0001

The primary endpoint was to investigate the association between hepatomiR^®^ P-scores and both PHLF (all grades A–C) and clinically relevant liver dysfunction (PHLF grades B and C). Here, significantly higher P-scores were documented in patients developing PHLF A–C as opposed to those without PHLF (Fig. 1A). In addition, likewise results could be observed for individuals with PHLF grades B and C (PHLF B + C) (Supplementary Fig. S1A). Further, receiver operating characteristics (ROC) curve analyses revealed good predictive potential for PHLF (A–C) with an area under the curve (AUC) of 0.770 and 0.755 for PHLF B + C, respectively (Fig. 1A).

### Commonly Used Preoperative Risk Assessment Tests Are Outperformed by HepatomiR^®^

In the next step, hepatomiR^®^ was compared with other established liver function tests. While APRI + ALBI was significantly higher in patients with PHLF A–C (Fig. 1B) and PHLF B + C (Supplementary Fig. S1A) compared with those without PHLF, ICG-clearance failed to distinguish between the two groups (PHLF: Fig. 1B; PHLF B + C: Supplementary Fig. S1A). ROC curve analyses confirmed that hepatomiR^®^ was superior to both tests in the prediction of PHLF A–C as well as PHLF B + C (Fig. 1C).

**Fig. 2:**
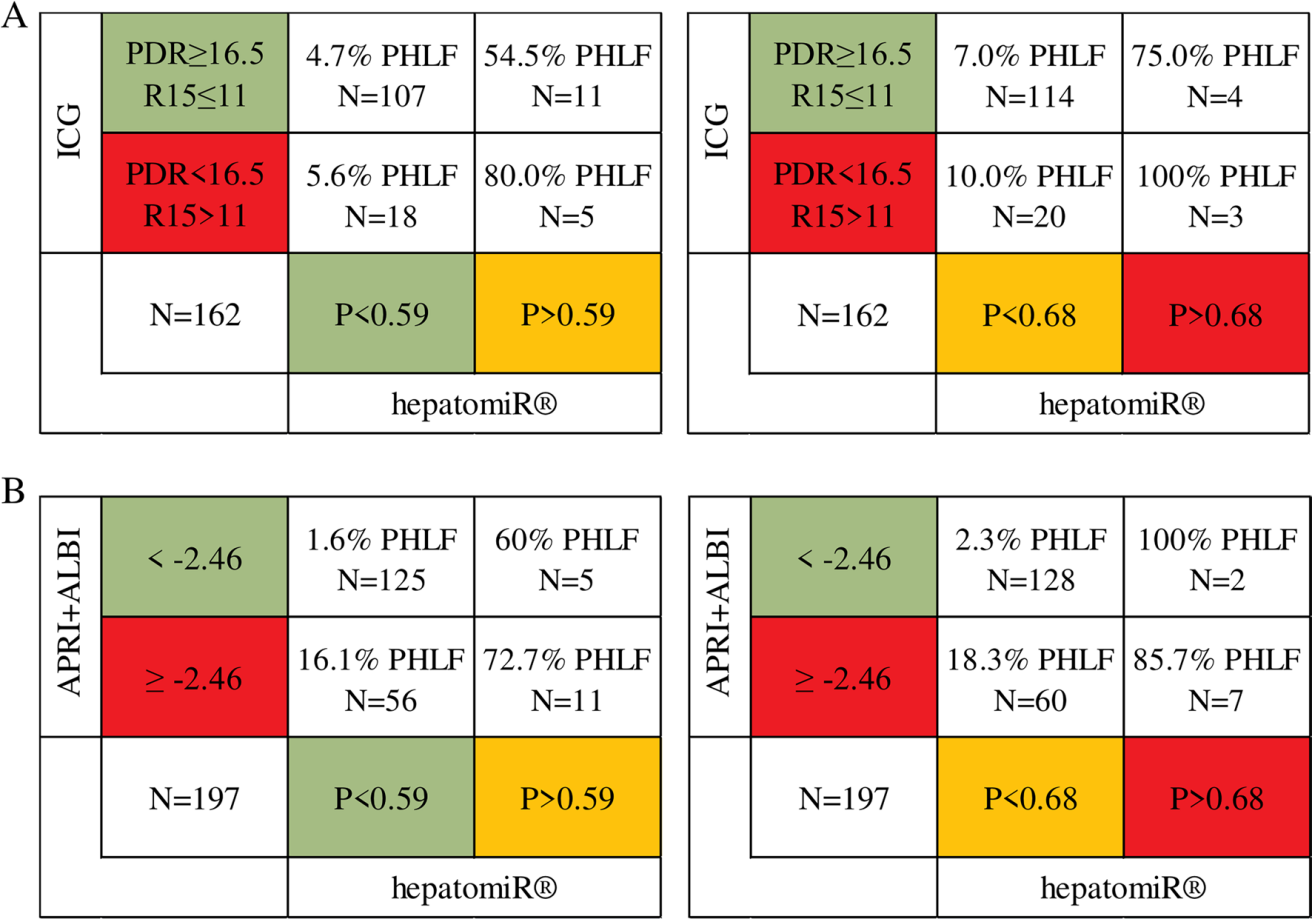
Risk groups of hepatomiR^®^ and other liver function tests compared: crosstabs illustrating the event of PHLF across different risk groups of hepatomiR^®^ and ICG-clearance (**A**)* and APRI + ALBI (**B**) cut-offs; * total number of patients who received ICG-clearance testing is included; *N* number, *APRI+ALBI* aspartate aminotransferase-to-platelet ratio index and albumin-bilirubin grade, *PHLF* posthepatectomy liver failure

Standard cut-offs for ICG-clearance^16^ and APRI + ALBI^17^ were applied and compared with hepatomiR^®^ risk groups. Among patients classified as having normal liver function based on ICG-clearance, 75% of those above the high-risk hepatomiR^®^ cut-off and 54.5% of those above the low-risk cut-off developed PHLF. In contrast, most patients classified as high-risk by ICG-clearance, could be appropriately reclassified by hepatomiR^®^ P-scores to low-risk (Fig. 2A). Similarly, hepatomiR^®^ cut-offs were able to detect patients with PHLF, who were incorrectly labelled by APRI + ALBI (Fig. 2B), highlighting the strong predictive potential of P-scores for PHLF.

### HepatomiR^®^ Accurately Defines High-Risk Patients

The NPV and PPV of cut-offs of hepatomiR^®^, as well as APRI + ALBI and ICG-clearance, were estimated. While the APRI + ALBI cut-off (≥ −2.46) had a marginally higher NPV than both hepatomiR^®^ cut-offs (P > 0.59; P > 0.68) for PHLF and PHLF B + C, the PPV of hepatomiR^®^ was considerably higher than that of all other tests (Table 2). Comparable results were obtained after stratifying patients by either major or minor resection (Supplementary Tables S3, S4).

**Table 2:** Negative and positive predictive values of liver function tests

PHLF	P > 0.59	P > 0.68	APRI + ALBI*	ICG^¥^	PHLF B + C	P > 0.59	P > 0.68	APRI + ALBI*	ICG^¥^
**PPV**	62.5%	77.0%	25.4%	21.7%	**PPV**	50.0%	61.5%	22.4%	21.7%
**NPV**	91.5%	90.2%	96.2%	90.7%	**NPV**	94.1%	96.4%	97.7%	95.0%

*APRI + ALBI* aspartate aminotransferase-to-platelet ratio index and albumin–bilirubin grade, *ICG* indocyanine green clearance, *NPV* negative predictive value, *PHLF* posthepatectomy liver failure, *PHLF B + C* PHLF grades B and C, *PPV* positive predictive value, *P > 0.59* hepatomiR^®^ low-risk cut-off, *P > 0.68* hepatomiR^®^ high-risk cut-off; * cut-off: −2.46; ¥ cut-off: PDR: 16.5, R15: 11

### Significant Differences in HepatomiR^®^ P-scores in Liver Disease Subgroups

**Fig. 3:**
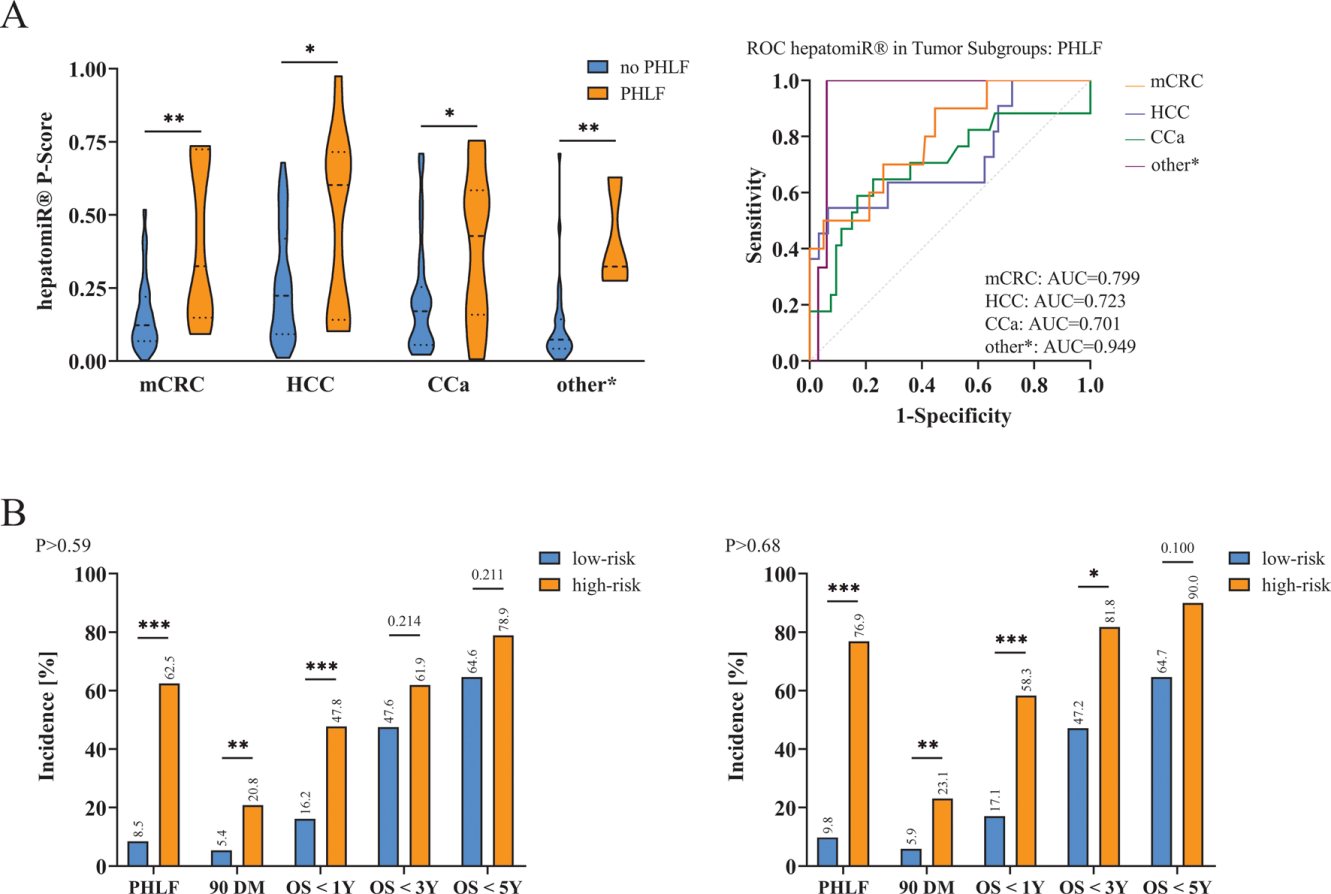
HepatomiR^®^ in tumor subgroups and overall survival: differences in hepatomiR^®^ without and with PHLF in the respective tumor subgroups, ROC curve analyses of different tumor subgroups (**A**), other* benign liver disease, and other malignancies; bar graphs depicting differences between low- and high-risk groups for the outcome parameters PHLF and overall survival (OS) up to 5 years across the low-risk cut-off P > 0.59 and the high-risk cut-off P > 0.68, the incidence of events in % is given on the *y*-axis (**B**); *AUC* area under the curve, *CCa* cholangiocellular carcinoma, *HCC* hepatocellular carcinoma, *mCRC* metastasized colorectal cancer, *OS* overall survival, *PHLF* posthepatectomy liver failure, *90 DM* 90-day mortality; * *p* < 0.05, ** *p* < 0.01, *** *p* < 0.0001

The risk for PHLF significantly varies between indications, based on the prevalence of underlying liver disease.^4^ P-scores were significantly higher in patients suffering from PHLF in comparison with patients who did not show any signs of PHLF in all entities of liver malignancies/diseases. To further assess the predictive potential of hepatomiR^®^ for PHLF within specific disease subgroups ROC curve analysis was performed, demonstrating reliable prediction of PHLF in all subgroups (Fig. 3A).

### HepatomiR^®^ Cut-Offs Are Predictive for OS up to 1 Year after Liver Resection

Previous studies have identified PHLF B + C as an independent risk factor for 90-day mortality.^18^ However, some patients will not develop fulminant hepatic failure with immediate postoperative death, but instead develop a state of chronically unstable liver function. These patients tend to die from hepatic failure at a later time-point after hepatic resection, usually during an infectious process.^19^ Hence, it was evaluated whether hepatomiR^®^ P-scores could also predict such delayed hepatic decompensation. 90-day mortality (90DM) was associated with higher hepatomiR^®^ P-scores prior to partial hepatectomy (Supplementary Fig. S1B). Further, a significantly higher number of patients died within 1 year when exhibiting a preoperative P-score above P > 0.59 or P > 0.68 (Fig. 3B). The differences in postoperative morbidity between patients are reported in the supplementary material (Supplementary Fig. S1C, D).

### Median Overall Survival Is Considerably Different across Low- and High-Risk Cut-Offs and Shows Tumor-Specific Differences

**Fig. 4:**
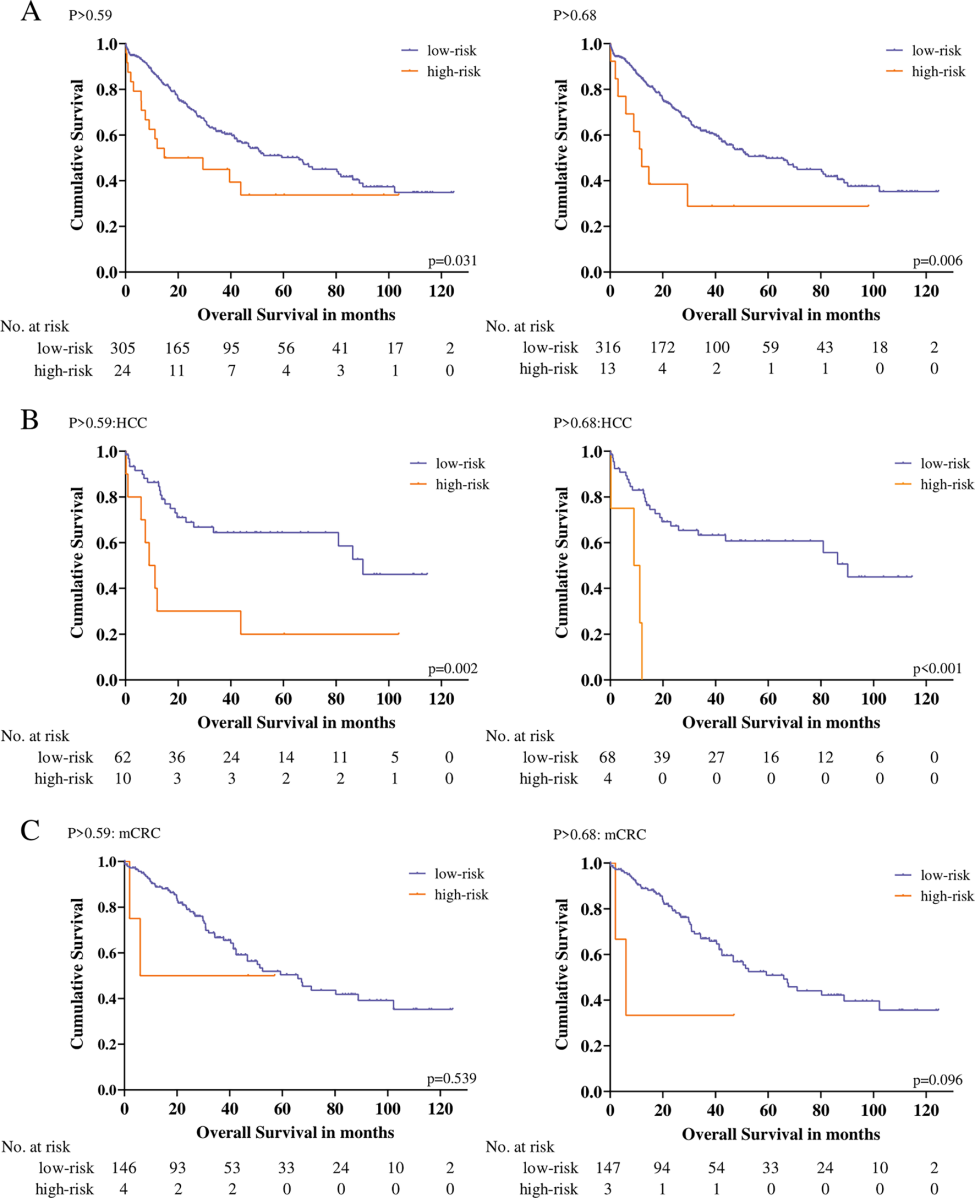
Overall survival differences between hepatomiR^®^ risk groups: Kaplan–Meier survival plots illustrating the difference in overall survival (OS) among both cut-offs, P > 0.59 and P > 0.68 (**A**), in the tumor subgroup HCC (**B**) and mCRC (**C**); the cumulative survival is plotted on the *y*-axis, the survival in months is plotted on the *x*-axis; a log-rank test was utilized to assess statistical differences; *HCC* hepatocellular carcinoma, *mCRC* metastasized colorectal cancer, *No.* number

The entire patient cohort was stratified according to the two cut-offs, P > 0.59 and P > 0.68,^15^ where significantly different median OS between risk groups were observed (Fig. 4A). In subgroup analysis by tumor type, patients with HCC in the high-risk groups had a tenfold shorter median OS compared with those in the low-risk groups for both cut-offs (Fig. 4B). Similarly, patients undergoing resection for mCRC showed a trend toward longer median OS in the low-risk group with the cut-off, P > 0.68 (Fig. 4C), while no differences were found in the CCa group. In addition, no associations between P-scores and progression-free survival (PFS) could be determined in the entire cohort or within individual tumor subgroups (Supplementary Table S5).

### Health and Budget Impact of the Uptake of HepatomiR^®^ for Preoperative Patient Stratification

HepatomiR^®^ stratified patients into three risk groups: low-risk, indicating immediate resection; medium-risk, indicating portal vein embolization to enhance liver regeneration prior to resection; and high-risk, indicating alternative cancer treatment as per the standard of care. Results evaluate the total number of PHLF for hepatomiR^®^ against the comparator actual standard of care, and direct costs for treatment including resection, medication, and treatment of PHLF.

In a base-case scenario, a 50% uptake rate of hepatomiR^®^ testing for risk assessments was assumed across the three different cancer types in Austria (462 patients tested per year), where it reduces PHLF incidence by 34 cases per year compared with the standard of care. With 80% uptake (594 patients), this number increases to 48 PHLF cases that can be avoided per year. Under these assumptions, the cost per avoided PHLF was 1309 € (HCC), 3731 € (CCa), and 3275 € (mCRC). To further evaluate the cost-effectiveness of hepatomiR^®^ screening, 1000 scenarios with varying assumptions for uptake rate, cost (material and personal), and incidence were calculated. It was found that 900 scenarios (90%) were within the limit of 10,000 € per avoided PHLF (Supplementary Fig. S2A, B).

A central point that shows the importance of risk management to reduce PHLF cases is the literature analysis of long-time survival of patients with HCC, mCRC, and CCa. The literature gives the basis for calculation of the life years gained (LYG) per reduced PHLF case.^20–22^ Using state of the art willingness to pay value of 50,000 €/LYG for direct costs in the Austrian setting, the hepatomiR^®^ test is cost-effective in 98.4% of all test scenarios (Supplementary Table S6).

## Discussion

As PHLF remains a major challenge in hepatobiliary surgery, this study presents an innovative test to improve preoperative risk assessment. This analysis confirms that the combination of three circulating miRs can predict PHLF and outperforms other liver function tests, such as ICG-clearance or APRI + ALBI. This applied to both immediate postoperative PHLF and postoperative survival up to 1 year, identifying patients who may derive only limited benefit from surgery. Also, a remarkably high PPV of 77% for the high-risk cut-off could be confirmed, clearly demonstrating clinical utility. Upon direct comparison of the cut-offs in relation to PHLF, hepatomiR^®^ exceeded the predictive potential of ICG-clearance. The use of hepatomiR^®^ was further supported by our healthcare economics analyses, indicating cost-effectiveness in 98.4% of patient scenarios. Taken together, the present data imply a strong predictive potential of miR-signatures for PHLF during preoperative risk stratification of patients undergoing hepatic resection.

miRs have become a strongly investigated area of research within the past decade, and have largely been described for their ability to act as biomarkers in various diseases, including primary and secondary liver tumors.^23–25^ They are short, non-coding RNAs that serve as invaluable regulators of cellular processes, and are characterized by a stable presence in bodily fluids.^26–28^ Moreover, a key advantage of utilizing a miR-based testing system is the mechanistic involvement in liver disease and function as opposed to common liver function tests that mainly reflect the liver’s elimination capacity. While the role of miRs in hepatic (patho-)physiology is yet to be fully understood, the miRs utilized in this study are well described. About 52% of the total hepatic miR copies are composed of miR-122, which is involved in crucial liver functions. It has been shown that miR-122 can be utilized as a biomarker for liver injury in chronic hepatitis B and C, as well as non-alcoholic steatohepatitis (NAFLD) or drug-induced liver injury (DILI).^29–31^ miR-192, which is also highly expressed in hepatocytes, has been described as an important marker of acute liver injury in a variety of liver diseases. Moreover, it was found to regulate inflammation in the progression of non-alcoholic steatohepatitis (NASH) by activating macrophages.^31^ In addition, the depletion of the cluster miR-192/194 promoted liver regeneration through the expression of β-catenin.^32^ miR-151 has been shown to be involved in the cell migration^33^ and in a recent study, miR-151, in contrast to miR-122, displayed lower plasma levels in patients diagnosed with HBV who had liver injury, but normal ALT levels,^34^ which might explain their differential regulation in patients suffering from PHLF.^15^

In addition to validating our previous findings that the hepatomiR^®^ P-score can predict PHLF and postoperative outcomes in general, the goal was to compare the performance of established liver function tests (ICG-clearance and APRI + ALBI).

ICG-clearance provides a dynamic assessment of liver function and has been applied regularly since its introduction. Despite its association with PHLF and postoperative mortality there are significant limitations, including the effects of changes in portal flow and cholestasis, making ICG-clearance infeasible for a significant number of patients.^16^ In contrast, hepatomiR^®^ was able to detect patients developing PHLF, who were not at risk according to ICG-clearance cut-offs.

Derived from routine laboratory parameters, the APRI + ALBI-score has previously been documented to predict PHLF as well as general postoperative outcomes after liver resection.^35–37^ While APRI was associated with chemotherapy-associated liver injury (CALI) after nCTx,^17^ ALBI alone has shown an association with fibrosis as well as cirrhosis in patients diagnosed with HCC, and was further correlated with survival and time to relapse.^38^ Of note, a multivariable model composed of APRI + ALBI, age, sex, tumor type, and the extent of resection, could predict PHLF B + C in different patient subgroups, and was equally reliable as the more expensive ICG-clearance.^11^ In this study however, hepatomiR^®^ demonstrated greater predictive potential than APRI + ALBI and ICG-clearance.

In our cohort, preoperative volumetry was available in 60 patients, where 7 of those had preoperative PVE. While some patients (*N* = 9) had a FLR below 40%, or even 30%, only one of these patients developed PHLF. From the remaining 51 patients with a FLR > 40%, 8 patients developed PHLF. This underlines the growing understanding that volumetric assessment of the FLR should be combined with liver function assessment. Of note, no patient was classified as intermediate- or high-risk by hepatomiR^®^ in the FLR < 40% group. This indicates that hepatomiR^®^ can identify patients at risk for PHLF even with a FLR > 40%. Further, as the P-scores could even identify patients at risk for PHLF undergoing only minor resections, the hypothesis that hepatomiR^®^ can unmask underlying (otherwise not identified) liver disease is reinforced. Interestingly, only one patient who had PVE was in the high-risk group of both cut-offs. However, we emphasize that basic principles of liver resection, such as the minimal required liver remnant, must still be respected.

The importance of personalized medicine has become increasingly recognized throughout the past years.^39,40^ A careful and precise assessment of liver function is a critical aspect of liver surgery, as there are still individuals with seemingly normal liver function who suffer from PHLF. This is highlighted by the fact that at least 41% of mortalities after hepatic surgery are directly linked to PHLF.^5,19^ In this context, the PPV of liver function tests is most important in the clinical setting, as it supports preoperative clinical decision making. The high-risk hepatomiR^®^ cut-off achieved a noteworthy PPV of 77% for PHLF and successfully recategorized patients who were initially inappropriately categorized by ICG-clearance and APRI + ALBI.

Certainly, our data indicate that hepatomiR^®^ contributes to preoperative risk assessment, as the combination with other liver function tests (such as APRI + ALBI or ICG-clearance) provided the best assessment of preoperative liver function. Indeed, our data nicely demonstrated that patients, classified as high-risk in multiple liver function tests (including hepatomiR^®^, were at the absolute highest risk of developing PHLF, even reaching up to 100% PPV when combining ICG and hepatomiR^®^ (Fig 2A, B).

Our data also suggest that the predominant driver for elevated P-scores is indeed liver function, as we observed no significant association with PFS. Particularly in HCCs, liver function and hepatic decompensation represent major determinants of patient OS. This likely explains why such a close association between the P-score with HCC OS was observed. Of note, an optimized liver function assessment in these patients could guide our treatment decisions in these patients—avoiding liver resection in patients that are unlikely to benefit from this strategy. This association with OS, however, was not evident in patients with CCa. CCa is a significantly more aggressive tumor type than HCC or mCRC, and early recurrence rates are much higher. Accordingly, tumor recurrences appear to be the major driver of postoperative OS, partially explaining why liver function (P-scores), while being predictive for PHLF, did not predict postoperative OS in this patient group.

In this study we developed and utilized a commercially available, CE-certified hepatomiR^®^ testing kit, which can be integrated into clinical routine by using real-time-quantitative PCR (RT-qPCR). Since the onset of COVID-19, RT-qPCR has emerged as the most widely utilized platform for molecular diagnostics, demonstrating remarkable scalability. Furthermore, the identification and stringent validation of key assay parameters have significantly enhanced its reproducibility, ensuring reliable and consistent results across diverse laboratory settings.^41^ These advancements, driven by the global need for rapid, high-throughput testing, have solidified RT-qPCR’s role as a gold standard in molecular diagnostics, including the quantification of miR biomarkers.

While the presented results validate previously published observations, some limitations must be addressed. In retrospective analyses, such as those presented here, results may be biased by the selection of patients or limited availability of data. Although no statistically significant differences in comorbidities were observed across the risk groups, it should be noted that these data were unavailable for some patients. Moreover, only patients deemed fit for surgery were included in this study; however, those with insufficient liver function for liver surgery are not represented. Moreover, volumetric analysis, although universally used in preoperative assessment, was only available in a small subgroup of patients. The discussed results would certainly benefit from a larger scale comparison with and in combination with liver volumetry. While 90DM and OS up to 1 year were closely associated with high-risk P-scores, a larger cohort of patients is required to more accurately assess short- and long-term postoperative mortality. Lastly, the health economic model only focused on common treatment strategies for the respective tumor subgroup. It is important to note that this approach does not fully capture the complexity of the clinical scenario and should instead be viewed as an illustrative example. To address these limitations, a prospective international multicenter study is needed, which is already in the process of being initiated. Here, hepatomiR^®^ will be further validated and categorized, aiming to define patient subgroups more effectively.

In conclusion, circulating miR-biomarkers such as hepatomiR^®^ have several key benefits, such as minimally invasive sample collection, short turnaround and scalability (RT-qPCR), and cost-effectiveness. However, its clear advantage lies in its ability to predict PHLF in a superior fashion compared with other established liver function tests. Moreover, concurrent use of the hepatomiR^®^ test with established clinical tests might give the most meaningful ability to predict patients at risk of developing PHLF. We provide two cut-offs, where patients below the low-risk cut-off are highly unlikely to develop PHLF in contrast to individuals above the high-risk cut-off, who are at particularly high risk. This association translated into significant effects on OS after resection, indicating that the P-score might allow us to select the ideal candidates for surgical resection and identify patients who may be better served with alternative treatments. While our results need further validation in larger cohorts of patients, we believe that they represent a cornerstone towards a new era of personalized patient assessment in hepatic surgery, to aid clinical decision making and make liver resections safer for our patients.

## Supplementary Information

Below is the link to the electronic supplementary material.

Supplementary file1 (DOCX 1776 KB)

## Data Availability

De-identified data can be made available to other researchers upon reasonable request.
