# Supplementary material for: MicroRNA based Prediction of Posthepatectomy Liver Failure and Mortality Outperforms Established Markers of Preoperative Risk Assessment
Source: Ann Surg Oncol. 2025 Jun 5;32(9):6283–94. doi: 10.1245/s10434-025-17528-x (PMC12317877; doi:10.1245/s10434-025-17528-x)
Supplement: Supplementary file 1 — Supplementary file1 (DOCX 1776 KB) [file 10434_2025_17528_MOESM1_ESM.docx]

**MicroRNA based Prediction of Posthepatectomy Liver Failure and Mortality Outperforms Established Markers of Preoperative Risk Assessment**

Anna Emilia Kern, MD^1^, David Pereyra, MD, PhD^2^, Jonas Santol, MD^3,4,5^, Markus Ammann, MD^4,6^, Sarang Kim, MD^1^, Felix Xaver Huber^1^, Jeremias Weninger^1^, Sarah Brunner^1^, Valerie Laferl^1^, Yannic Herrmann^1^, Anna Jankoschek^1^, Gregor Ortmayr, MD^7^, Benedikt Rumpf, MD^8^, Marcel Schuetze, MD^9^, Rosmarie Valenta, MD^9^, Christian Krestan, MD^9^, Guenther Zauner, PhD^10,11^, Melanie Zechmeister, BSc^10,12^, Susanna Skalicky, MSc^13^, Matthias Hackl, PhD^13^, Alice Assinger, PhD^14^, Thomas Gruenberger, MD^3^, Patrick Starlinger, MD, PhD^2,4,14^

1 Medical University of Vienna, Vienna, Austria
2 Department of General Surgery, Division of Visceral Surgery, Medical University of Vienna, Vienna, Austria
3 Department of Surgery, HPB Center, Vienna Health Network, Clinic Favoriten and Sigmund Freud Private University, Vienna, Austria
4 Department of Surgery, Division of Hepatobiliary and Pancreas Surgery, Mayo Clinic, Rochester, MN, USA
5 Institute of Vascular Biology and Thrombosis Research, Center for Physiology and Pharmacology, Medical University of Vienna, Vienna, Austria
6 Department of Surgery, State Hospital Wiener Neustadt, Wiener Neustadt, Austria
7 Center for Cancer Research, Medical University of Vienna, Vienna, Austria
8 Department of Surgery, Hospital Barmherzige Schwestern, Vienna, Austria
9 Department of Diagnostic and Interventional Radiology, Vienna Health Network, Clinic Favoriten, Vienna, Austria
10 DWH GmbH, Vienna, Austria
11 Institute of Information Systems Engineering, TU Wien, Vienna, Austria
12 Institute of Statistics and Mathematical Methods in Economics, TU Wien, Vienna, Austria
13 TAmiRNA GmbH, Vienna, Austria
14 Center for Physiology and Pharmacology, Medical University of Vienna, Vienna, Austria

**Corresponding author.** Patrick Starlinger, MD, PhD, Mayo Clinic, Rochester, 200 First St. SW, Rochester, MN 55905, USA

**Supplementary Materials - Index**

| **Supplementary Methods** |  |
| --- | --- |
| Study population | *pag. 3* |
| Measurement of routine blood parameters  Assessment of Preoperative Liver Function  Definition and classification of posthepatectomy liver failure and postoperative morbidity  qPCR analysis of circulating miRs  Health Economic Modelling  Postoperative Follow-up  Statistical Analyses | *pag. 3*  *pag. 3*  *pag. 3-4*  *pag. 4*  *pag. 4-5*  *pag. 5*  *pag. 5* |
| **Supplementary Results** |  |
| Postoperative Outcome | *pag. 6* |
| **Supplementary Figures and Tables** |  |
| Figure S1 | *pag. 7* |
| Figure S2  Table S1  Table S2  Table S3  Table S4  Table S5  Table S6 | *pag. 8*  *pag. 9*  *pag. 10*  *pag. 11*  *pag. 11*  *pag. 12*  *pag. 12* |
| **References** | *pag. 13* |
|  |  |

**Supplementary Methods**

**Study Population**

The study population consists of a heterogenic patient collective suffering from different liver diseases and all receiving major and minor liver surgery. 151 of 329 patients were diagnosed with mCRC (synchronous and metachronous metastasis), while 72 suffered from HCC. Of 70 patients with CCa, 45 had intrahepatic CCa, 13 were diagnosed with perihilar CCa, 11 had gallbladder cancer and one patient was diagnosed with a distal CCa. Moreover, 18 patients received resection due to benign liver disease, where two patients had adenomas, 5 had echinococcal cysts, three were diagnosed with focal nodular hyperplasia, hemangioma was present in 6 individuals and lastly two patients were diagnosed with cholangitis. Finally, 18 patients were diagnosed with other malignant liver diseases. Here, one patient had a mixed-type HCC/CCa, one was diagnosed with metastasis of a choriocarcinoma and one with metastasis of an anal carcinoma. Further, 4 patients hat liver metastasis of breast cancer, 6 with metastasis of neuroendocrine tumors and finally 5 patients with metastasized pancreatic cancer. All forms of underlying malignant and benign liver disease were confirmed at final pathology of the specimen retrieved upon resection.

**Measurement of routine blood parameters**

Platelet counts, serum bilirubin (SB), prothrombin time (PT), alcalic phosphatase (AP), gamma-glutamyltransferase (GGT), aspartate aminotransferase (AST), alanine aminotransferase (ALT) and albumin were measure in appropriate samples by routine laboratory tests.

**Assessment of Preoperative Liver Function**

Preoperative liver function was routinely assessed using indocyanine green (ICG)-clearance and the combined aspartate aminotransferase (AST)-to-platelet ratio index (APRI) and albumin-bilirubin grade (ALBI). For the assessment of liver function by analyzing the ICG-clearance, 25mg of ICG reagent were dissolved in 20ml of isotonic fluid and a dose of 0.25mg/kg of body weight was intravenously administered to the patient. The concentration of the color reagent in the circulation was then assessed using pulse spectrometry. Here, the amount of ICG reagent cleared within the first minute (=plasma disappearance rate (PDR)), and the remaining amount of reagent detected in the circulation after 15 minutes (=retention rate at 15 minutes (R15)), were measured. For the evaluation of sensitivity, specificity as well as positive and negative predictive values, ICG-clearance values were further dichotomized according to previously published cut-offs.

**Definition and classification of posthepatectomy liver failure and postoperative morbidity**

PHLF was defined according to the criteria published by the International Study Group of Liver Surgery (ISGLS).^1^ Here, PHLF is present if serum bilirubin levels and international standardized ratio on or after POD5 were abnormal. If patients had serum bilirubin levels as well as INR within the normal range before POD5, or were discharged early due to good clinical performance, they were considered to not have PHLF. Accordingly, PHLF was classified into grades A-C, where no change of clinical management was required for patients suffering from grade A PHLF, while those with grade C PHLF needed to undergo invasive treatment. To evaluate postoperative complications, the classification published by Dindo et al.^2^ was used. Patients who died within 90 days after surgery were considered to have postoperative mortality.

**qPCR analysis of circulating miRs**

Blood was drawn preoperatively into pre-cooled CTAD tubes and processed within 30 minutes of acquisition. Plasma was meticulously prepared as previously.^3^ In brief, after sample acquisition the plasma was separated from solid blood components via centrifugation at 1 000 g and 4°C for 10 minutes, followed by an additional centrifugation step for 10 minutes at 10 000 g and 4°C. Finally, plasma was stored at -80°C until further analysis.

Total RNA was extracted from 200 µl of platelet-poor plasma using the Plasma RNA extraction kit included with the hepatomiR® CE-IVD test kit (component 1/3). Total RNA was eluted in 30 µl nuclease free water and either analyzed directly or stored at -80°C until further analysis. Reverse transcription and qPCR analysis was performed using the hepatomiR® chemistry kit (component 2/3) and hepatomiR® 384-well plates (component 3/3). RT-qPCR reactions were performed using the Roche LightCycler 480 II instrument using a two-step PCR protocol according to the product manual. The kit measures 8 different assays per sample, of which 5 are quality controls: hsa-miR-451a and hsa-miR-23a-3p serve as indicators of sample hemolysis. Based on the miR-23°-3p/451a ratio, a cut-off of <7 was used to exclude hemolytic samples. RNA-, cDNA, and PCR-spike-ins are used to monitor the analytical variability and exclude samples with insufficient RNA extraction efficiency or RT-qPCR analysis from further analysis.

The hepatomiR® software application (v.1.0) was used to first calculate the Cq-values from the fluorescent data on the basis of second derivative maximum method. Cq-values obtained from hsa-miR-122-5p, hsa-miR-151a-5p, and hsa-miR-192-5p were used to calculate the delta Cq-values for two ratios: miR-122-5p/miR-151a-5p and miR-151a-5p/miR-192-5p (termed self-normalizing pairs). These delta Cq-values were used to derive the hepatomiR® P-Score on the basis of a logistic regression models, whose parameters had been previously defined.^4^ Calculated hepatmiR® P-Scores were then utilized for further analyses discussed in this manuscript.

**Health Economic Modelling**

In the standard-of-care scenario (without hepatomiR®) the direct costs were calculated by considering the cost for a resection for every patient. In the scenario with hepatomiR® the costs of three patient-groups stratified by hepatomiR® were added together:

• Low-risk patients (P<0.59): Cost of a resection for every patient

• Medium-risk patients (P>0.59-P<0.68): Cost of embolization for every patient plus costs for a resection for 90% of these patients plus cost of alternative therapy for the remaining 10% of the patients

• High-risk patients (P>0.69): Cost of alternative therapy for every patient

In Austria inpatient hospital stays are grouped into procedure-oriented diagnosis-related case flat rates (LDFs). The procedures are defined in,^5^ the related flat rates can be found in.^6^ According to this the cost for a resection in Austria were defined by considering the procedures HL032 and HL042. For the embolization, all the procedures are matched to MEL 20. The cost for the alternative therapy for the three cancer types were calculated by the following procedures:

• CRCLM: FOLFOX (procedure SC588) and Bevacizumab (XA060)

• CCC: Cisplatin/Gemcitabine (XC312)

• HCC: Radiofrequency ablation (HL010)

**Postoperative Follow-up**

All patients within our biobank were followed up postoperatively as per clinical routine. Hence, every three months within the first two years after surgery and subsequently, the intervals were extended to six months until year five, and yearly thereafter. Follow up consisted of clinical examinations, cross-sectional imaging (CT and MRI) and routine laboratory testing, which included assessment of tumor markers (CEA, CA 19-9). During this period, apart from overall survival (OS), disease-free-survival (DFS) was also assessed. Here DFS was considered between the day of surgery and the first evidence of tumor recurrence according to available radiological imaging results and tumor board consensus. In this patient collective, the median follow up was 20 months (range: 0.1-124.7 months).

**Statistical Analyses**

Statistical analyses were carried out using the Software IBM SPSS Statistics 27.0.1 (IBM Corp., Armonk, NY) and were based on nonparametric tests, receiver operating characteristics (ROC) analysis, and log-rank tests. GraphPad Prism 8 (GraphPad Software, La Jolla, CA) and Microsoft Office 16 Word and Excel were used for drawing figures and tables. P-values <0.05 were regarded as statistically significant. To compare parameters between two groups, Mann-Whitney-U-Test was utilized. Overall and progression free survival were analyzed by Kaplan-Meier survival analysis.

**Supplementary Results**

**Postoperative Outcome**

Patients suffering from postoperative morbidity had higher P-Scores preoperatively compared to patients without (*p=0.035*) (Fig. S1C). In addition, patients suffering from severe postoperative morbidity (Clavien-Dindo ≥ grade 3) also presented with higher preoperative P-Scores (*p<0.001*) (Fig. S1D).

**Supplementary Figures and Tables**

**
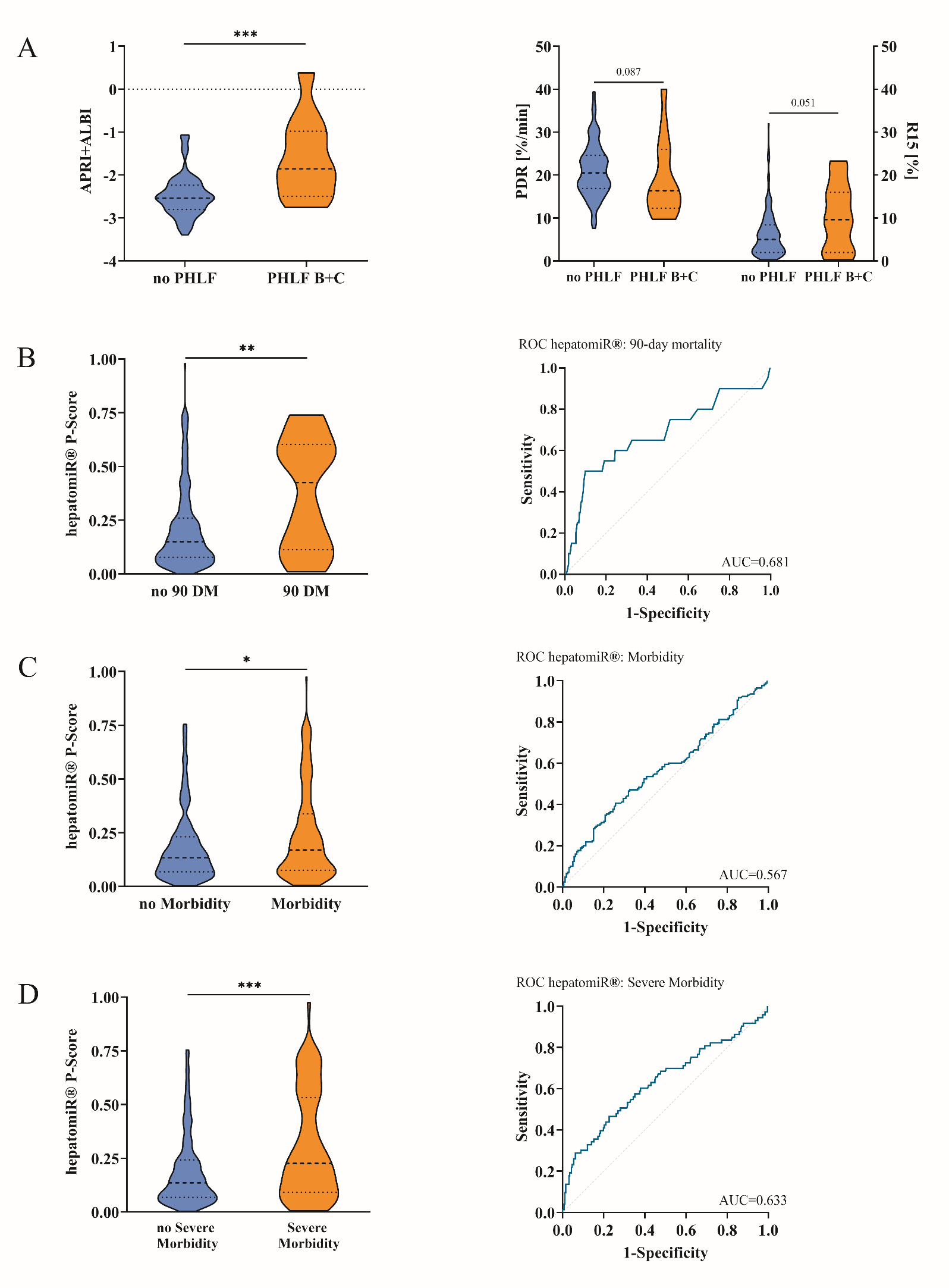
**

**Figure S1.** Violin Plots comparing APRI+ALBI-Scores, and ICG-clearance (B) among patients without and with PHLF grades B and C. Comparison of hepatomiR® P-Scores between patients without and with 90 day mortality (B), morbidity (C) and severe morbidity (D) as well as the affiliated ROC curves.

**
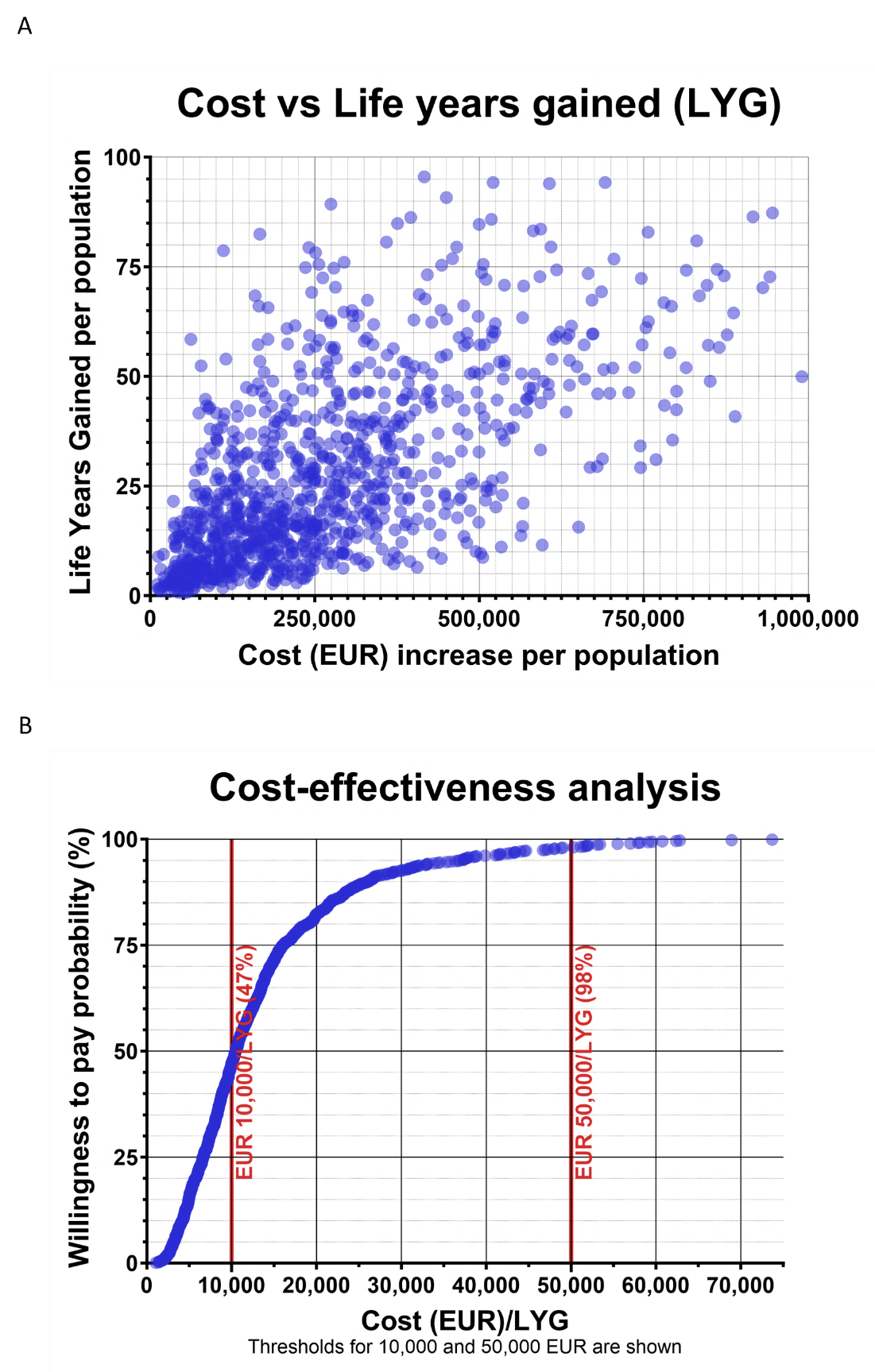
**

**Figure S2.** hepatomiR® cost-effectiveness analysis. (A) The total cost impact in EUR for the population is plotted against the life-year gained (LYG) for 1000 different scenarios. (B) The probability (%) for cost effectiveness based on 1000 scenarios is shown. Threshold for EUR 10,000/LYG and EUR 50,000/LYG are highlighted. More than 98% of scenarios estimate additional cost of EUR 50,000 or less per LYG.

| **Table S1.** | | | | | | | |
| --- | --- | --- | --- | --- | --- | --- | --- |
|  | **Low-Risk (N=305)** | **High-Risk  (N=24)** |  |  | **Low-Risk (N=305)** | **High-Risk**  **(N=24)** |  |
| **Parameter** | **Median (Range)/N (%)** | | **p-value** | **Parameter** | **Median (Range)/N (%)** | | **p-value** |
| **Gender** |  |  | 0.302 | **Co-Morbidities** |  |  |  |
| Male | 197 (64.6) | 18 (75) |  | Hypertension | 110 (53.1) | 12 (60) | 0.557 |
| Female | 108 (35.4) | 6 (25) |  | Type 2 Diabetes | 36 (17.4) | 4 (20) | 0.760 |
| **Age (years)** | 65 (22-90) | 68 (35-82) | 0.204 | Renal Insufficiency | 15 (4.9) |  |  |
| **Resection Type** |  |  |  | Pulmonary Disease | 18 (8.7) |  |  |
| Minor | 139 (45.6) | 4 (16.7) | 0.005 | **Morbidity** |  |  | <0.001 |
| Major | 166 (54.5) | 20 (83.3) | 0.005 | No Morbidity | 152 (49.8) | 7 (29.2) |  |
| Open Surgery | 219 (92) | 21 (100) | 0.379 | Grade I | 25 (8.2) | 0 (0.0) |  |
| MIS | 19 (8.0) |  |  | Grade II | 70 (23) | 3 (12.5) |  |
| **FLR<30%*** | 3 (5.0) |  |  | Grade III | 35 (11.5) | 8 (33.3) |  |
| **FLR<40%*** | 9 (15) |  |  | Grade IV | 8 (2.7) | 2 (8.3) |  |
| **Tumor Type** |  |  |  | Grade V | 15 (4.9) | 4 (16.7) |  |
| mCRC | 147 (48.2) | 4 (16.7) | 0.003 | **PHLF ISGLS** |  |  | <0.001 |
| HCC | 62 (20.3) | 10 (41.7) | 0.021 | No PHLF | 279 (91.5) | 9 (37.5) |  |
| CCa | 62 (20.3) | 8 (33.3) | 0.191 | ISGLS A | 8 (2.6) | 3 (12.5) |  |
| Other | 17 (5.6) | 1 (4.2) | 1.000 | ISGLS B | 7 (2.3) | 2 (8.3) |  |
| Benign | 17 (5.6) | 1 (4.2) | 1.000 | ISGLS C | 11 (3.6) | 4 (16.7) |  |
| **Co-Factors** |  |  |  | **Postop. Stay** |  |  |  |
| nCTx | 106 (34.8) | 3 (12.5) | 0.462 | ICU (days) | 1 (0-56) | 3 (0-15) | <0.001 |
| PVE | 16 (15.2) | 1 (4.2) | 0.692 | Total Hosp. (days) | 7 (1-87) | 15 (6-117) | <0.001 |
| Steatosis (%) | 5 (0-100) | 5 (0-80) | 0.974 | **mCRC** |  |  |  |
| Steatohepatitis | 5 (19) | 9 (37.5) | 0.019 | synchrone Mets. | 75 (68.8) | 1 (25) | 0.102 |
| Intraoperative RBCs | 20 (6.5) | 8 (33.3) | <0.001 | metachrone Mets. | 34 (31.2) | 3 (75) | 0.102 |
| **Fibrosis** | 183 (60) | 15 (62.5) | 0.271 | **nCTx regimen** |  |  |  |
| Grade I | 105 (34.4) | 5 (20.8) |  | FOLFOX | 5 (4.7) |  |  |
| Grade II | 35 (11.5) | 2 (12.5) |  | FOLFOX+Avastin | 15 (14.2) |  |  |
| Grade III | 21 (6.9) | 2 (8.3) |  | FOLFOX+Cetuximab | 2 (1.9) |  |  |
| Grade IV | 22 (7.2) | 5 (20.8) |  | FOLFOX+Panitumumab | 4 (3.8) |  |  |
| **Preop. Parameters** |  |  |  | FOLFIRI+Avastin | 4 (3.8) |  |  |
| hepatomiR® P-Scores | 0.137 (0.002-0.584) | 0.686 (0.595-0.980) | <0.001 | FOLFIRI+Cetuximab | 5 (4.7) | 1 (33.3) | 0.158 |
| PDR (%/mL) | 20.4 (7.6-39.4) | 18.7 (9.9-40) | 0.581 | FOLFIRI+Panitumumab | 5 (4.7) |  |  |
| R15 (%) | 5.0 (0.3-32) | 7.6 (0.3-22.7) | 0.393 | FOLFOXIRI | 4 (3.8) |  |  |
| APRI+ALBI | -2.62 (-5.6- -0.62) | -2.10 (-3.26-0.38) | 0.002 | FOLFOXIRI+Avastin | 13 (12.3) |  |  |
| Platelets (10^3^/µL) | 236 (71-1082) | 227 (86-383) | 0.209 | FOLFOXIRI+Cetuximab | 1 (0.9) | 1 (33.3) | 0.055 |
| SB (mg/dL) | 0.54 (0.1-20.37) | 0.87 (0.15-2.22) | 0.009 | FOLFOXIRI+Panitumumab | 1 (0.9) |  |  |
| PT (%) | 105 (45-150) | 100 (40-137) | 0.088 | FOLFIRINOX+Avastin | 2 (1.9) |  |  |
| AP (U/L) | 90 (32-1274) | 104 (45-707) | 0.104 | XELOX | 3 (2.8) |  |  |
| GGT (U/L) | 55 (9-2457) | 194 (19-1576) | <0.001 | XELOX+Avastin | 26 (24.1) |  |  |
| AST (U/L) | 31 (13-418) | 44 (22-224) | 0.009 | XELOX+Cetuximab | 4 (3.8) |  |  |
| ALT (U/L) | 28 (7-806) | 47 (12-617) | <0.001 | XELOX+Panitumumab | 3 (2.8) |  |  |
| Albumin (g/L) | 41.9 (28.1-74.2) | 42.2 (31.5-48.5) | 0.623 | XELIRI+Avastin | 3 (2.8) |  |  |
|  |  |  |  | XELIRI+Cetuximab | 1 (0.9) | 1 (33.3) | 0.055 |
|  |  |  |  | XELIRI+Panitumumab | 1 (0.9) |  |  |
| **Patient demographics: P>0.59.** ALT=alanine aminotransferase, AP=alcalic phosphatase, APRI+ALBI=aspartate aminotransferase-to-platelet ratio index and albumin-bilirubin grade, AST=aspartate aminotransferase, CCa=cholangiocellular carcinoma, FLR=future liver remnant, FOLFOX=Folinic acid/5-fluoruracil/Oxaliplatin, FOLFIRI= Folinic acid/5-fluoruracil/Irinotecan, FOLFOXIRI= Folinic acid/5-fluoruracil/Oxaliplatin/Irinotecan, FOLFIRINOX= Folinic acid/5-fluoruracil/Irinotecan/Oxaliplatin, GGT=gamma-glutamyl transpeptidase, HCC=hepatocellular carcinoma, ICU=intensive care unit, ISGLS=International Study Group of Liver Surgery, mCRC=metastasized colorectal cancer, mets.=metastases, N=Number, nCTx=neoadjuvant chemotherapy, PDR=plasma disappearance rate, PHLF=Posthepatectomy liver failure, preop.=preoperative, postop.=postoperative, PT=prothrombin time, PVE=portal vein embolization, R15=retention rate at 15 minutes, RBCs=red blood cells, SB=serum bilirubin, XELOX=Capecitabine/Oxaliplatin, XELIRI=Capecitabine/Irinotecan, * preoperative volumetry was available in 60 patients | | | | | | | |

| **Table S2.** | | | | | | | |
| --- | --- | --- | --- | --- | --- | --- | --- |
|  | **Low-Risk (N=316)** | **High-Risk (N=13)** |  |  | **Low-Risk (N=316)** | **High-Risk (N=13)** |  |
| **Parameter** | **Median (Range)/N (%)** | | **p-value** | **Parameter** | **Median (Range)/N (%)** | | **p-value** |
| **Gender** |  |  | 0.232 | **Co-Morbidities** |  |  |  |
| Male | 204 (64.6) | 11 (84.6) |  | Hypertension | 116 (53.7) | 6 (54.5) | 0.956 |
| Female | 112 (35.4) | 2 (15.4) |  | Type 2 Diabetes | 39 (18.1) | 1 (9.1) | 0.694 |
| **Age (years)** | 65 (22-90) | 68 (35-82) | 0.331 | Renal Insufficiency | 15 (6.9) |  |  |
| **Resection Type** |  |  |  | Pulmonary Disease | 18 (8.3) |  |  |
| Minor | 141 (44.6) | 2 (15.4) | **0.037** | **Morbidity** |  |  | 0.016 |
| Major | 175 (55.4) | 11 (84.6) | **0.037** | No Morbidity | 156 (49.4) | 3 (23.1) |  |
| Open Surgery | 229 (92.3) | 11 (100) | 1.000 | Grade I | 25 (7.9) | 0 (0.0) |  |
| MIS | 19 (7.7) |  |  | Grade II | 72 (22.8) | 1 (7.7) |  |
| **FLR<30%*** | 3 (5.0) |  |  | Grade III | 37 (11.7) | 6 (46.2) |  |
| **FLR<40%*** | 9 (15.0) |  |  | Grade IV | 9 (2.8) | 1(7.7) |  |
| **Tumor Type** |  |  |  | Grade V | 17 (5.4) | 2 (15.4) |  |
| mCRC | 148 (46.8) | 3 (23.1) | 0.092 | **PHLF ISGLS** |  |  | <0.001 |
| HCC | 68 (21.5) | 4 (30.8) | 0.492 | No PHLF | 285 (90.2) | 3 (23.1) |  |
| CCa | 65 (20.6) | 5 (38.5) | 0.160 | ISGLS A | 9 (2.8) | 2 (15.4) |  |
| Other | 18 (5.7) | 0 (0.0) | 0.526 | ISGLS B | 8 (2.5) | 1 (7.7) |  |
| Benign | 17 (5.4) | 1 (7.7) | 1.000 | ISGLS C | 14 (4.4) | 7 (53.8) |  |
| **Co-Factors** |  |  |  | **Postop. Stay** |  |  |  |
| nCTx | 86 (27.2) | 2 (15.4) | 0.370 | ICU (days) | 1 (0-56) | 3 (0-15) | <0.001 |
| PVE | 16 (14.3) | 1 (12.5) | 1.000 | Total Hosp. (days) | 8 (1-117) | 20 (7-50) | <0.001 |
| Steatosis (%) | 5 (0-100) | 7 (0-50) | 0.752 | **mCRC** |  |  |  |
| Steatohepatitis | 62 (19.6) | 5 (38.5) | 0.053 | synchrone Mets. | 75 (68.2) | 1 (33.3) | 0.249 |
| Intraoperative RBCs | 24 (7.6) | 4 (30.8) | 0.021 | metachrone Mets. | 35 (31.8) | 2 (66.7) | 0.249 |
| **Fibrosis** | 189 (59.8) | 9 (69.2) | 0.482 | **nCTx regimen** |  |  |  |
| Grade I | 105 (33.2) | 5 (38.5) |  | FOLFOX | 5 (4.7) |  |  |
| Grade II | 35 (11.1) | 3 (23.1) |  | FOLFOX+Avastin | 15 (14) |  |  |
| Grade III | 23 (7.3) | 0 (0.0) |  | FOLFOX+Cetuximab | 2 (1.9) |  |  |
| Grade IV | 26 (8.2) | 1 (7.7) |  | FOLFOX+Panitumumab | 4 (3.7) |  |  |
| **Preop. Parameters** |  |  |  | FOLFIRI+Avastin | 4 (3.7) |  |  |
| hepatomiR® P-Scores | 0.143 (0.002-0.679) | 0.734 (0.684-0.980) | <0.001 | FOLFIRI+Cetuximab | 6 (5.6) |  |  |
| PDR (%/mL) | 20 (7.6-40) | 20 (9.9-26.7) | 0.489 | FOLFIRI+Panitumumab | 5 (4.7) |  |  |
| R15 (%) | 5.0 (0.3-32.0) | 9.6 (1-22.7) | 0.239 | FOLFOXIRI | 4 (3.7) |  |  |
| APRI+ALBI | -2.61 (-5.62 - 0.38) | -2.10 (-2.76 - -0.56) | 0.005 | FOLFOXIRI+Avastin | 13 (12.1) |  |  |
| Platelets (10^3^/µL) | 234 (71-1082) | 228 (155-383) | 0.775 | FOLFOXIRI+Cetuximab | 1 (0.9) | 1 (50) | 0.037 |
| SB (mg/dL) | 0.54 (0.1-20.37) | 0.93 (0.49-2.22) | 0.002 | FOLFOXIRI+Panitumumab | 1 (0.9) |  |  |
| PT (%) | 105 (45-150) | 79 (40-119) | 0.007 | FOLFIRINOX+Avastin | 2 (1.9) |  |  |
| AP (U/L) | 89 (32-1274) | 160 (65-300) | 0.016 | XELOX | 3 (2.8) |  |  |
| GGT (U/L) | 57 (9-2457) | 205 (53-708) | <0.001 | XELOX+Avastin | 26 (23.9) |  |  |
| AST (U/L) | 31 (13-418) | 54 (22-224) | 0.014 | XELOX+Cetuximab | 4 (3.7) |  |  |
| ALT (U/L) | 29 (7-806) | 56 (25-617) | <0.001 | XELOX+Panitumumab | 3 (2.8) |  |  |
| Albumin (g/L) | 41.9 (28.1-74.2) | 40.7 (31.5-45.3) | 0.178 | XELIRI+Avastin | 3 (2.8) |  |  |
|  |  |  |  | XELIRI+Cetuximab | 1 (0.9) | 1 (50) | 0.037 |
|  |  |  |  | XELIRI+Panitumumab | 1 (0.9) |  |  |
| **Patient demographics: P>0.68.** ALT=alanine aminotransferase, AP=alcalic phosphatase, APRI+ALBI=aspartate aminotransferase-to-platelet ratio index and albumin-bilirubin grade, AST=aspartate aminotransferase, CCa=cholangiocellular carcinoma, FLR=future liver remnant, FOLFOX=Folinic acid/5-fluoruracil/Oxaliplatin, FOLFIRI= Folinic acid/5-fluoruracil/Irinotecan, FOLFOXIRI= Folinic acid/5-fluoruracil/Oxaliplatin/Irinotecan, FOLFIRINOX= Folinic acid/5-fluoruracil/Irinotecan/Oxaliplatin, GGT=gamma-glutamyl transpeptidase, HCC=hepatocellular carcinoma, ICU=intensive care unit, ISGLS=International Study Group of Liver Surgery, mCRC=metastasized colorectal cancer, mets.=metastases, N=Number, nCTx=neoadjuvant chemotherapy, PDR=plasma disappearance rate, PHLF=Posthepatectomy liver failure, preop.=preoperative, postop.=postoperative, PT=prothrombin time, PVE=portal vein embolization, R15=retention rate at 15 minutes, RBCs=red blood cells, SB=serum bilirubin, XELOX=Capecitabine/Oxaliplatin, XELIRI=Capecitabine/Irinotecan, * preoperative volumetry was available in 60 patients | | | | | | | |

| **Table S3.** | | | | | | | | | |
| --- | --- | --- | --- | --- | --- | --- | --- | --- | --- |
| Major hepatectomy | | | | | | | | | |
| **P>0.59** | **NPV** | **PPV** | **Sen.** | **Spec.** | **P>0.68** | **NPV** | **PPV** | **Sen.** | **Spec.** |
| **PHLF** | 84.9% | 65.0% | 34.2% | 95.3% | **PHLF** | 82.9% | 72.7% | 21.1% | 98.0% |
| **PHLF B+C** | 89.8% | 50.0% | 37.0% | 93.7% | **PHLF B+C** | 88.0% | 54.5% | 22.2% | 96.9% |
| Minor hepatectomy | | | | | | | | | |
| **P>0.59** | **NPV** | **PPV** | **Sen.** | **Spec.** | **P>0.68** | **NPV** | **PPV** | **Sen.** | **Spec.** |
| **PHLF** | 99.3% | 50.0% | 66.7% | 98.6% | **PHLF** | 99.3% | 100% | 66.7% | 100% |
| **PHLF B+C** | 99.3% | 50.0% | 66.7% | 98.6% | **PHLF B+C** | 99.3% | 100% | 66.7% | 100% |
| **NPV, PPV, sensitivity and specificity of hepatomiR® in major and minor hepatectomy subgroups.** NPV=negative predictive value, PHLF=Posthepatectomy liver failure, PHLF B+C=PHLF grades B and C, PPV=positive predictive value, P>0.59=hepatomiR® low-risk cut-off, P>0.68=hepatomiR® high-risk cut-off, Sen.=Sensitivity, Spec.=Specificity | | | | | | | | | |

| **Table S4.** | | | | |
| --- | --- | --- | --- | --- |
| Major hepatectomy | | | | |
| **PDR ≥16.5 + R15≤11** | **NPV** | **PPV** | **Sen.** | **Spec.** |
| **PHLF** | 84.9% | 45.5% | 31.3% | 91.2% |
| **PHLF B+C** | 91.8% | 45.5% | 45.5% | 91.8% |
| Minor Hepatectomy | | | | |
| **PDR ≥16.5 + R15≤11** | **NPV** | **PPV** | **Sen.** | **Spec.** |
| **PHLF** | 100% | 0% | 100% | 78.9% |
| **PHLF B+C** | 100% | 0% | 100% | 78.9% |
| **NPV, PPV, sensitivity and specificity of ICG-clearance in major and minor hepatectomy subgroups.** ICG=indocyanine green, NPV=negative predictive value, PDR=plasma disappearance rate, PHLF=Posthepatectomy liver failure, PHLF B+C=PHLF grades B and C, PPV=positive predictive value, R15=retention rate at 15 minutes, Sen.=Sensitivity, Spec.=Specificity | | | | |

| **Table S5.** | | | | | |
| --- | --- | --- | --- | --- | --- |
| **Entire Cohort** | | | | | |
| **P>0.59** | **Events/N (%)** | **Median PFS (95% CI in months)** | **P>0.68** | **Events/N (%)** | **Median PFS (95% CI in months)** |
| **low-risk** | 167/251 (66.5) | 14.1 (10.4-18.8) | **low-risk** | 170/260 (65.4) | 14.9 (11.3-18.4) |
| **high-risk** | 11/22 (50.0) | 19.2 (7.6-30.8) | **high-risk** | 8/13 (61.2) | 8.7 (0.3-17.0) |
| **Log-Rank** | *p=0.615* |  | **Log-Rank** | *p=0.531* |  |
| **HCC** | | | | | |
| **P>0.59** | **Events/N (%)** | **Median PFS (95% CI in months)** | **P>0.68** | **Events/N (%)** | **Median PFS (95% CI in months)** |
| **low-risk** | 35/54 (64.8) | 26.3 (14.9-37.8) | **low-risk** | 36/60 (60.0) | 31.7 (19.3-44.1) |
| **high-risk** | 3/10 (30.0) | 12.6 (3.3-21.8) | **high-risk** | 2/4 (50.0) | 8.1 |
| **Log-Rank** | *p=0.341* |  | **Log-Rank** | *p=0.367* |  |
| **mCRC** | | | | | |
| **P>0.59** | **Events/N (%)** | **Median PFS (95% CI in months)** | **P>0.68** | **Events/N (%)** | **Median PFS (95% CI in months)** |
| **low-risk** | 96/127 (75.6) | 11.1 (9.2-13.1) | **low-risk** | 97/128 (75.8) | 11.2 (9.2-13.1) |
| **high-risk** | 3/4 (75.0) | 19.2 (0.0-45-7) | **high-risk** | 2/3 (66.7) | 2.6 |
| **Log-Rank** | *p=0.853* |  | **Log-Rank** | *p=0.587* |  |
| **CCa** | | | | | |
| **P>0.59** | **Events/N (%)** | **Median PFS (95% CI in months)** | **P>0.68** | **Events/N (%)** | **Median PFS (95% CI in months)** |
| **low-risk** | 26/48 (54.2) | 30.1 (14.1-38.5) | **low-risk** | 27/50 (54.0) | 28.1 (12.0-44.2) |
| **high-risk** | 5/7 (71.4) | 14.4 (0.0-37.7) | **high-risk** | 4/5 (80.0) | 14.4 (2.1-26.6) |
| **Log-Rank** | *p=0.130* |  | **Log-Rank** | *p=0.806* |  |
| **Progression free survival: entire cohort and tumor subgroups.** CCa=cholangiocellular carcinoma, CI=confidence intervall, HCC=hepatocellular carcinoma, mCRC=metastasized colorectal cancer, N=number, PFS=progression free survival, P>0.59=hepatomiR® low-risk cut-off, P>0.68=hepatomiR® high-risk cut-off | | | | | |

| **Table S6.** | | | | | | | | |
| --- | --- | --- | --- | --- | --- | --- | --- | --- |
| **HCC** | **1 year** | **3 years** | **5 years** | **≤ 1 year** | **1-3 years** | **3-5 years** | **Lost years** | **Difference** |
| **with PHLF** | 69% | 45% | 45% | 31% | 24% | 0% | 2.11 |  |
| **without PHLF** | 94% | 73% | 58% | 6% | 21% | 15% | 1.07 | 1.04 |
| **mCRC** | **1 year** | **3 years** | **5 years** | **≤ 1 year** | **1-3 years** | **3-5 years** | **Lost years** | **Difference** |
| **with PHLF** | 86% | 65% | 42% | 14% | 21% | 23% | 1.49 |  |
| **without PHLF** | 95% | 66% | 45% | 5% | 29% | 21% | 1.31 | 0.19 |
| **CCa** | **1 year** | **3 years** | **5 years** | **≤ 1 year** | **1-3 years** | **3-5 years** | **Lost years** | **Difference** |
| **with PHLF** | 67% | 41% | 21% | 33% | 26% | 20% | 2.48 |  |
| **without PHLF** | 83% | 45% | 28% | 17% | 38% | 17% | 2.07 | 0.41 |
| **Life-years gained per reduced PHLF case in different tumor subgroups.** PHLF=Posthepatectomy liver failure | | | | | | | | |

**References**

1. Rahbari NN, Garden OJ, Padbury R, et al. Posthepatectomy liver failure: a definition and grading by the International Study Group of Liver Surgery (ISGLS). *Surgery*. May 2011;149(5):713-24. doi:10.1016/j.surg.2010.10.001

2. Dindo D, Demartines N, Clavien PA. Classification of surgical complications: a new proposal with evaluation in a cohort of 6336 patients and results of a survey. *Ann Surg*. Aug 2004;240(2):205-13. doi:10.1097/01.sla.0000133083.54934.ae

3. Starlinger P, Alidzanovic L, Schauer D, et al. Platelet-stored angiogenesis factors: clinical monitoring is prone to artifacts. *Dis Markers*. 2011;31(2):55-65. doi:10.3233/dma-2011-0798

4. Starlinger P, Hackl H, Pereyra D, et al. Predicting Postoperative Liver Dysfunction Based on Blood-Derived MicroRNA Signatures. *Hepatology*. Jun 2019;69(6):2636-2651. doi:10.1002/hep.30572

5. Bundesministerium für Soziales G, Pflege und Konsumentenschutz (BMSGPK). Modell 2024 - Anlage 2, Medizinische Einzelleistungen mit zugeordneten MEL-Gruppen. 2024;

6. Bundesministerium für Soziales G, Pflege und Konsumentenschutz (BMSGPK). Modell 2023 - Anlage 5, LDF-Baumdarstellung. 2023;
